# Exposure to Antibiotics Affects Saponin Immersion-Induced Immune Stimulation and Shift in Microbial Composition in Zebrafish Larvae

**DOI:** 10.3389/fmicb.2018.02588

**Published:** 2018-10-29

**Authors:** Adrià López Nadal, David Peggs, Geert F. Wiegertjes, Sylvia Brugman

**Affiliations:** ^1^Cell Biology and Immunology, Animal Sciences Group, Wageningen University and Research, Wageningen, Netherlands; ^2^Skretting Animal Research Centre, Stavanger, Norway; ^3^Aquaculture and Fisheries, Animal Sciences Group, Wageningen University and Research, Wageningen, Netherlands

**Keywords:** saponin, zebrafish, microbiota, oxytetracycline, ciprofloxacin, neutrophils, macrophages

## Abstract

In the last decades, pollution of the environment by large scale use of antibiotics in agriculture and human medicine have led to increased antimicrobial resistance in both the environment and the host animal microbiome. Disturbances in the host microbiome can result in impaired immunity and reduced resilience of aquaculture species. Here, we investigated whether environmentally measured levels of the commonly used antibiotics ciprofloxacin and oxytetracycline influences the host microbiome and susceptibility toward saponin-induced immune stimulation in larval zebrafish. Firstly, neutrophil and macrophage reporter zebrafish larvae were exposed to different concentrations of soy saponin by immersion. A dose-dependent increase in neutrophil presence in the intestinal area was observed together with increased expression of immune genes *il1b, tnfa, il22* and *mmp9*. To investigate the effect of antibiotics, larval zebrafish were immersed in ciprofloxacin or oxytetracycline in the presence or absence of a low dose of saponin. *In vivo* imaging revealed that antibiotic treatment did not reduce the number of neutrophils that were recruited to the intestinal area upon saponin exposure, although it did tend to lower pro-inflammatory cytokine levels. Microbial sequencing of whole larvae revealed that exposure to a low dose of saponin already shifted the microbial composition. The combination of oxytetracycline and saponin significantly increased α-diversity compared to the controls. In conclusion, the current study provides evidence that the combination of low levels of antibiotics with low levels of anti-nutritional factors (saponin) can induce inflammatory phenotypes and can modify the microbiota, which might lead to altered disease susceptibility.

## Introduction

With a growing world population, reaching an estimated 9 billion people in the year 2050, the need for food to feed the world is a pressing matter. Aquaculture is one of the fastest growing production sectors globally, and fish consumption increased from 9.9 kg/capita in the 1960s on average to a staggering 19.7 kg in 2013 and it is estimated to grow further. Aquaculture is now surpassing captured fisheries and amounts to 90 million tons of farmed fish worldwide (FAO report, SOFIA, 2016). For sustainable fish production to meet global demand now and in the future, performance and sustainability of fish feeds should be improved both from an economic and ecological stand point.

The development of high-quality sustainable aquaculture feed is a challenge due to the varying availability of raw materials that ensure sufficient protein levels and the presence of unhealthy anti-nutritional factors. For example, in the past, costly fish meal was replaced by cheaper plant-based feeds such as soybean meal (Sales, 2009). However, soybean meal may contain large amounts of anti-nutritional compounds, such as soy saponin. Soy saponin has been consistently shown to induce enteritis and alter the microbiome composition in farmed fish (Chikwati et al., 2012; Costas et al., 2014; Krogdahl et al., 2015). More recently, a multitude of feed sources have become available such as peas, faba bean and rapeseed meals, as well as highly refined concentrates. As the feed industry adapts to account for new ingredient sources, appropriate *in vivo* models are required to test feeds for their effects on fish health prior to large scale production.

Next to the importance of feed for fish health, the fish environment (water, sediment, and plants) contains many microbes that can both be beneficial as well as detrimental to their health (Bentzon-Tilia et al., 2016; de Bruijn et al., 2018). Beneficial bacteria can help digest feed as well as reduce the level of toxic metabolites (such as ammonia or nitrate) ensuring good water quality (Bentzon-Tilia et al., 2016). However, the large amounts of antibiotics that have been used as growth promoters in animal husbandry and human medicine are posing a threat to our health and those of our aquaculture fish (Ding and He, 2010; Klein et al., 2018). Large scale antibiotic resistance and the rise of opportunistic infections calls for multidisciplinary research efforts to increase the resilience of all species including aquaculture fish (Watts et al., 2017).

In a recent review of the European scenario it was shown that the levels of antibiotic in the water can reach as high as several micrograms per liter (Carvalho and Santos, 2016). These antibiotics, besides inducing antimicrobial resistance (Gullberg et al., 2011; Pindling et al., 2018), also have an impact on the fish microbial communities of gut, skin and gills, which in turn influences fish disease susceptibility (Navarrete et al., 2008; Brugman et al., 2009; Tacchi et al., 2015; Pindling et al., 2018; Zhou et al., 2018a,b). For example, previously, we showed that adult zebrafish exposed to a high dose (mg/L range) of the antibiotic vancomycin showed an overgrowth of *Cetobacterium somerae* (a fish commensal) and displayed reduced severity of chemically induced enterocolitis, whereas fish exposed to a high dose (mg/L range) of colistin sulphate showed overgrowth of *Aeromonas sp.* and were not protected from enterocolitis (Brugman et al., 2009). Furthermore, Zhou et al. (2018a) exposed adult zebrafish to low dose (ng/L range) antibiotics sulfamethoxazole or oxytetracycline for a 6-week period and reported an increased metabolic rate and higher *Aeromonas hydrophila*-induced mortality. Gut function of these zebrafish was impaired as evidenced by a decrease in intestinal goblet cell numbers, alkaline phosphatase and acid phosphatase activity. Furthermore an increased expression of pro-inflammatory cytokines *tnfα* and *il1* was observed (Zhou et al., 2018a).

Given the potential for antibiotic treatments to alter host responses to antigens such as anti-nutritional factors in the feed, there is a need to understand whether reported low (ng/L – μg/L) environmental concentrations of antibiotics might change the microbial composition, which in turn might influence disease susceptibility. In this study, we set-up an immersion-based saponin immune stimulation model using zebrafish larvae. Subsequently, we addressed whether environmentally encountered levels (μg/L range) of the antibiotics ciprofloxacin or oxytetracycline influence the saponin-induced immune stimulation and the fish microbiome.

## Materials and Methods

### Ethics Statement

The present study was approved by the Dutch Committee on Animal Welfare (2017.W-0034) and the Animal Welfare Body (IvD) of the Wageningen University (Netherlands). Furthermore, we adhere to our standard biosecurity and institutional safety procedures at Wageningen University and Research.

### Animals

Adult Tg(mpeg1:mCherry/mpx:eGFPi^114^) (Renshaw et al., 2006; Bernut et al., 2014) zebrafish (kindly provided by Prof. Meijer, Leiden University), expressing mCherry under the macrophage-specific mpeg1 promotor and GFP under the neutrophil-specific mpx promotor were housed in Zebtec family tanks (Tecniplast, Buguggiate, Italy) under continuous flow-through at 28°C (14/10-hour light/dark cycle) at Carus facilities (WUR, Wageningen, Netherlands). Zebrafish were fed with a mixture of Artemia 230.000 npg (Ocean Nutrition Europe, Essen, Belgium) and Tetramin Flakes (Tetra, Melle, Germany) twice per day. Embryos were obtained by natural spawning and raised with E3 water (0.10 mM NaCl in demineralized water, pH 7.6) in petri dishes at 28°C (12/12-hour light/dark cycle) (Westerfield, 2007). Dead or fungus-infected embryos were identified by microscopy and discarded in tricaine/E3 solution [8.4% (v/v) 24 mM Tricaine (Sigma-Aldrich, DL, United States) stock solution in E3]. Larval ages are expressed in days post-fertilization (dpf). From 5 dpf onward larvae were fed with live daily cultured *Tetrahymena pyriformis.*

### Dose-Response Experiment Saponin Exposure

Double Tg(mpeg1:mCherry/mpx:eGFPi^114^) zebrafish larvae were randomly distributed in 6 well plates (*n* = 20 fish/well) and exposed to different concentrations [0, 0.5, 0.7 and 1.0 mg/ml] of saponin [ultrapure Soy Saponin 95%, kindly provided by Trond Kortner NMBU Oslo Norway, origin: Organic Technologies, Coshocton, OH (Krogdahl et al., 2015)] dissolved in the E3 (10 ml solution/well) from 6–9 dpf. Mortality was registered and all media were refreshed daily. At 24 h (7 dpf) and 72 h (9 dpf) after the start of the immersion, zebrafish (*n* = 6–11/group) were anaesthetized embedded and imaged using fluorescent microscopy (as described below). Per time point several larvae were euthanized for further analysis with an overdose MS-222 (8.4 ml of 24 mM Tricaine (Sigma-Aldrich, DL, United States) in 100 ml E3). Pools of 5 larvae were used for RNA extraction (3 pools per group at 24 h, 7–9 pools per group at 72 h) and gene expression was measured on cDNA by Real Time PCR (as described below). Two independent experiments were performed and data were combined.

### Experimental Design and Sampling Strategy Antibiotics and Saponin Exposure

**FIGURE 1:**
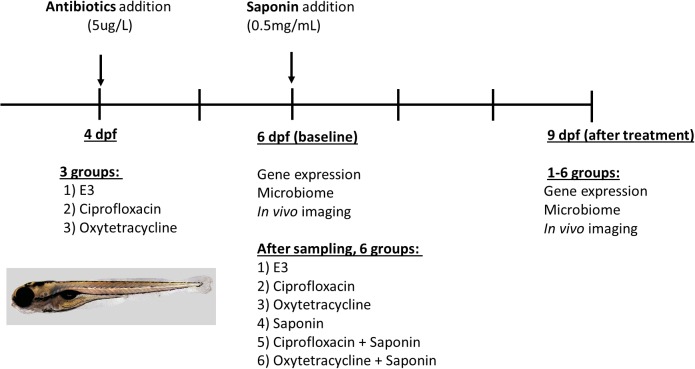
Experimental design and sampling strategy for the antibiotics/saponin experiment.

A graphical representation of the experimental design and analysis performed per time-point is displayed in Figure 1. To assess the effect of antibiotics, 4 dpf Tg(mpeg1:mCherry/mpx:eGFPi^114^) fish were randomly distributed in five 6 well-plates (*n* = 20 fish/well) and 3 treatment conditions were established: (1) control (E3), (2) ciprofloxacin 5 μg/L (Sigma-Aldrich, DL, United States) or (3) oxytetracycline hydrochloride 5 μg/L (Sigma-Aldrich, DL, United States) (10 ml solution/well). The dose of antibiotics was based on several reviews and experimental papers summarizing environmental concentrations of antibiotics in water environments (Ding and He, 2010; Carvalho and Santos, 2016; Watts et al., 2017; Patrolecco et al., 2018; Zhou et al., 2018b) to be at a low dose (ng-μg/L range) and not acute dose (mg/L range). At 6 dpf, 4 pools of 5 larvae were sampled to assess changes in gene expression at baseline. Moreover, at 6 dpf DNA was isolated from 3 pools of 5 larvae to investigate microbiome composition at baseline. *In vivo* imaging was performed on *n* = 10 larvae/group to visualize innate immune cells. Subsequently, after sampling, at 6 dpf ultrapure soy saponin was applied to half of the remaining larvae at a concentration 0.5 mg/ml (to induce mild immune stimulation) so each treatment group was split into two, resulting in 6 treatment groups: (1) control, (2) ciprofloxacin (5 μg/L), (3) oxytetracycline hydrochloride (5 μg/L), (4) saponin (0.5 mg/ml), (5) ciprofloxacin + saponin (5 μg/L + 0.5 mg/ml), and (6) oxytetracycline hydrochloride + saponin (5 μg/L + 0.5 mg/ml). All treatment media were refreshed daily. At 9 dpf *in vivo* imaging was performed on *n* = 10 larvae/group to visualize innate immune cells. Gene expression was performed on 4 pools of 5 larvae to investigate immune gene expression and from 3 pools of 5 larvae DNA was isolated for microbiological analysis.

### Fluorescent *in vivo* Imaging

Tg(mpeg1:mCherry/mpx:eGFPi^114^) zebrafish larvae were anaesthetized with tricaine/E3 solution (4.2 ml of 24 mM Tricaine (Sigma-Aldrich, DL, United States) in 100 ml E3) and embedded in 1% low melting point agarose (Thermo Fisher Scientific, MA, United States). Larvae were imaged as whole mounts with a Leica M205 FA Fluorescence Stereo Microscope. After image acquisition, pictures were analyzed with ImageJ^®^ software (United States National Institutes of Health, Bethesda, United States). The intestinal regions were manually selected per fish on the basis of the bright light picture and subsequently copied to the green and red channel pictures (Supplementary Figure S1). Within this intestinal region individual cells were counted for each fish. Furthermore, corrected total cell fluorescence (CTCF) was measured in ImageJ^®^ on total fish larvae by using the following formula: Integrated density–(area of total fish x mean fluorescence of the background reading).

### Relative Gene Expression

In order to assess changes in gene expression, total RNA was isolated from pools of larvae (*n* = 5/pool) with the RNeasy^®^ Mini Kit (QIAGEN, Hilden, Germany) according to the manufacturer’s instructions. The RNA concentration was assessed with the NanoDrop 1000 Spectrophotometer (Thermo Fisher Scientific, MA, United States). The quality of RNA was assessed by analysis of the 260/280 (1.9–2.0) and 260/230 (2.0–2.2) ratio on the nanodrop. cDNA was synthetized including a DNase treatment [DNase I (1 U/μl)], followed by synthesis using Superscript^TM^ III First Strand Synthesis Systems (Invitrogen, CA, United States). Finally, Real-Time quantitative Polymerase Chain Reaction (RT-qPCR) was performed with the use of ABsolute^TM^ qPCR SYBR^®^ Green Mix (Thermo Fisher Scientific, MA, United States) using the Thermal cycler Rotor-Gene 6000^TM^ (Corbett Research, Cambridge, United Kingdom). The PCR program used was the following: 95°C 3 min. 40 × (95°C 10 s, 60°C 10 s, 72°C 30 s) followed by a melting curve 95°C 30 s, 65°C 5 s increase to 95°C in 0.5°C steps. Data were normalized to *elf1α* and the Pfaffl quantification method with efficiency correction (Pfaffl, 2001) was applied as described in Forlenza et al. (2012). The primers used are listed in Table 1.

**Table 1:** Forward (FW) and reverse (RV) sequences of the primers employed for the Real Time qPCR.

Gene	FW primer	RV primer
*elfα1*	5′-CTGGAGGCCAGCTCAAACAT-3′	5′-ATCAAGAAGAGTAGTAGTACCG-3′
*il1*β	5′-TGCGGGCAATATGAAGTCA-3′	5′-TTCGCCATGAGCATGTCC-3′
*cxcl8a*	5′-TGTTTTCCTGGCATTTCTGACC-3′	5′-TTTACAGTGTGGGCTTGGAGGG-3′
*ccl2*	5′-GTCTGGTGCTCTTCGCTTTC-3′	5′-TGCAGAGAAGATGCGTCGTA-3′
*il22*	5′-GGAGGGTCTGCACAGAG-3′	5′-GTCTCCCCGATTGCTT-3′
*tnfα2 (tnfb)*	5′-AAACAACAAATCACCACACC-3′	5′-ACACAAAGTAAAGACCATCC-3′
*mmp9*	5′-ACGGCATTGCTGACAT-3′	5′-TAGCGGGTTTGAATGG-3′
*il10*	5′-AGGGCTTTCCTTTAAGACTG-3′	5′-ATATCCCGCTTGAGTTCC-3′

### 16S rRNA Gene Profiling

In order to study the microbiome composition total DNA was isolated from three pools of 5 larvae per treatment condition and time-point (6 and 9dpf). Samples were kept in 2 ml Eppendorf^®^ tubes with 100 μl lysis buffer (100 nM NaCl, 10 nM Tris pH∼8, 15 nM EDTA, 0.5% w/v SDS) and 7 μl of Proteinase K (19 mg/ml) (QIAGEN, Hilden, Germany). Samples were incubated at 56°C until dissolved. Subsequently, 35 μl of a saturated 6 M NaCl solution was added, leaving the samples on a shaker for 15 min. After centrifugation at 21000 × *g* for 15 min the DNA-containing supernatants were transferred to a new tubes and 270 μl of ice-cold 100% ethanol was added. Samples were incubated at −20°C for 10 min and after 5 min. centrifugation at 21000 × *g* 15°C, the pellet was washed and dissolved in 50 μl of RNase-free water. A DNA clean-up step was performed using DNA Clean & Concentrator^TM^ kit (Zymo Research, CA, United States) following manufacturer’s instructions. DNA concentration was measured using the NanoDrop 1000 Spectrophotometer (Thermo Fisher Scientific, MA, United States). Samples below 100 ng per sample, 260/280 ratio ≤ 1.80 and 260/280 ratio ≤ 1.50 were excluded from further analysis.

Illumina 16S rRNA gene amplicon libraries were generated and sequenced at BaseClear (Leiden, Netherlands). In short, barcoded amplicons from the V3–V4 region of 16S rRNA genes were generated using a 2-step PCR. 10–25 ng genomic (g)DNA was used as template for the first PCR with a total volume of 50 μl using the 341F (5′-CCTACGGGNGGCWGCAG-3′) and the 785R (5′-GACTACHVGGGTATCTAATCC-3′) primers appended with Illumina adaptor sequences. Control PCR reactions were performed alongside each separate amplification without addition of template. PCR products were purified and the size of the PCR products were checked on Fragment analyzer (Advanced Analytical) and quantified by fluorometric analysis. Purified PCR products were used for the 2nd PCR in combination with sample-specific barcoded primers (Nextera XT index kit, Illumina). Subsequently, PCR products were purified, checked on a Fragment analyzer (Advanced Analytical) and quantified, followed by multiplexing, clustering, and sequencing on an Illumina MiSeq with the paired-end (2×) 300-bp protocol and indexing. The sequencing run was analyzed with the Illumina CASAVA pipeline (v1.8.3) with demultiplexing based on sample-specific barcodes. The raw sequencing data produced was processed removing the sequence reads of too low quality (only “passing filter” reads were selected) and discarding reads containing adaptor sequences or PhiX control with an in-house filtering protocol. A total number of ∼588.000 reads were distributed in ∼22.000 reads per sample on average. In addition, reads containing (partial) adapters were clipped (up to a minimum read length of 50-bp.). A quality assessment on the remaining reads was performed using the FASTQC quality control tool version 0.10.0. The Illumina paired reads were merged into single reads (so-called pseudoreads) through sequence overlap with SNAP version 1.0.23 (Naccache et al., 2014), after removal of the forward and reverse primers (Edgar, 2010). Chimeric pseudoreads were removed and the remaining reads were aligned to the RDP 16S gene databases (Cole et al., 2014). Based on the alignment scores of the pseudoreads, the taxonomic depth of the lineage is based on the identity threshold of the rank; Species 99%, Genus 97%, Family 95%, Order 90%, Class 85%, and Phylum 80%. A total number of ∼105.000 high-quality, paired-end, unique reads were clustered into 578 OTUs. These OTUs were further filtered excluding the ones contributing ≤ 0.01% of the dataset resulting in 239 OTUs. An overview of the control quality measurements for the samples is displayed in Table 2.

**Table 2:** Control quality measurements for all the samples: pseudo-reads, classified reads, coverage percentage and number of observed OTUs per sample.

Samples	Pseudo-reads	Classified reads	Coverage percentage	Observed OTUs n°/sample
(1) Control 6 dpf	22302	21427	97.1	99
(2) Control 6 dpf	23513	22716	98.7	76
(3) Control 6dpf	23296	22335	98.2	83
(4) Ciprofloxacin 6 dpf	20581	19661	98.2	96
(5) Ciprofloxacin 6 dpf	27550	26536	98.2	76
(6) Ciprofloxacin 6 dpf	27275	26246	98.0	122
(7) Oxytetracyclin 6 dpf	17427	16873	97.7	52
(8) Oxytetracyclin 6 dpf	21250	20580	97.7	65
(9) Oxytetracyclin 6 dpf	19794	19120	98.1	127
(10) Control 9 dpf	21635	20902	97.4	62
(11) Control 9 dpf	26136	25222	97.9	64
(12) Control 9 dpf	16466	15979	97.7	68
(13) Saponin 9 dpf	24279	23397	96.3	149
(14) Saponin 9 dpf	21079	20401	97.8	133
(15) Saponin 9 dpf	18037	17327	97.9	123
(16) Ciprofloxacin 9 dpf	21892	21183	97.9	84
(17) Ciprofloxacin 9 dpf	19922	19282	97.6	68
(18) Ciprofloxacin 9 dpf	21037	20319	97.6	96
(19) Ciprofloxacin + Saponin 9 dpf	19005	18269	97.7	125
(20) Ciprofloxacin + Saponin 9 dpf	20790	20044	98.0	103
(21) Ciprofloxacin + Saponin 9 dpf	20382	19681	98.3	115
(22) Oxytetracyclin 9 dpf	19808	19185	97.4	114
(23) Oxytetracyclin 9 dpf	17192	16646	97.6	104
(24) Oxytetracyclin 9 dpf	19497	18839	97.9	93
(25) Oxytetracyclin + Saponin 9 dpf	24147	23125	96.9	161
(26) Oxytetracyclin + Saponin 9 dpf	21400	20566	98.2	174
(27) Oxytetracyclin + Saponin 9 dpf	19352	18108	98.1	154

### Statistics

The data collected from the fluorescent imaging of the saponin dose response was analyzed using Prism version 5.03 (GraphPad^©^); linear regression and one-way ANOVA with *post hoc* test after confirmation of normal distribution of the data (Kolmogorov-Smirnov test). The α-diversity graphs, as well as the relative gene expression and the innate immune cells counts were generated in Prism version 5.03 (GraphPad^©^). The former plots were firstly tested with D’Agostino and Pearson omnibus normality algorithms and further analyzed with either one/two-way(s) Analysis of Variance (ANOVA) and Tukey’s Post-test or Kruskall-Wallis test and Dunn’s Multiple Comparison Post-test for normal and non-normal distributed data, respectively.

The dataset containing 239 abundance-standardized OTUs was employed to further establish relationships among bacterial communities. Data was rarefied using MicrobiomeAnalyst^©^ (Dhariwal et al., 2017) and α-diversity indexes including Observed OTUs, Shannon index, Simpson index, Chao1 and Fisher index were calculated accordingly. In order to assess β-diversity, Principal Coordinate Analysis (PCoA) plots were derived from unweighted UniFrac and Bray-Curtis dissimilarity distances by permutational multivariate analysis of variance (PERMANOVA) using MicrobiomeAnalyst^©^. Furthermore, Redundancy Analysis (RDA) plots were assessed by using Canoco^©^ 5.0 (Canoco version 5.0, Braak, C.J.F. ter; Smilauer, P Microcomputer Power) in order to correlate microbial communities with the treatments. These analyses were based on Bray-Curtis dissimilarity distances and assessed using permutational multivariate analysis of variance (PERMANOVA).

## Results

### Dose-Dependent Increase of Neutrophil Recruitment to the Intestinal Region After Three Days of Saponin Exposure

**FIGURE 2:**
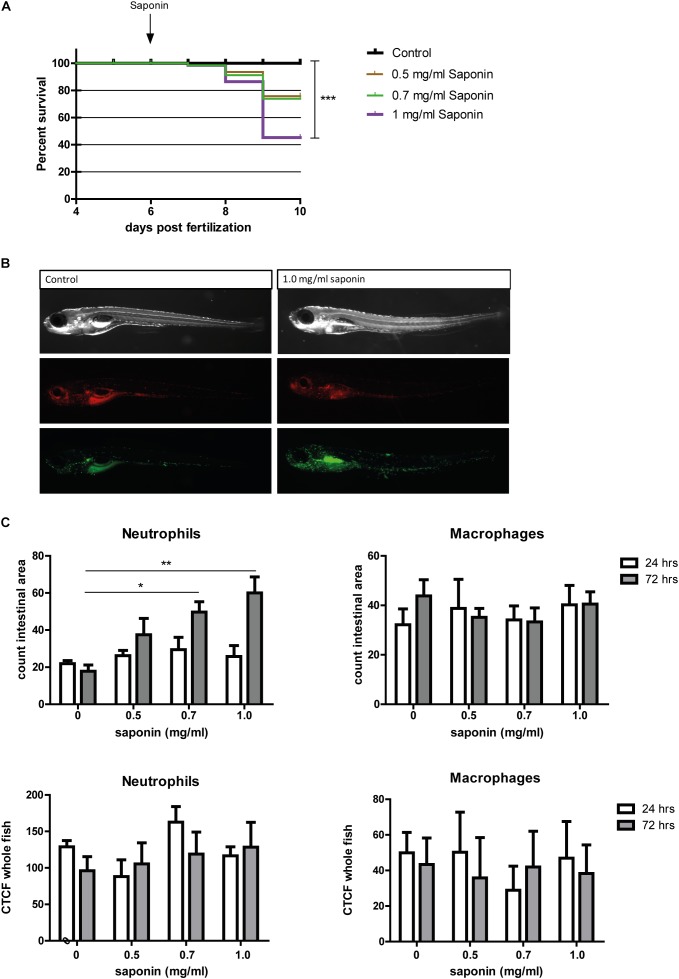
Effect of saponin immersion on zebrafish larvae. **(A)** Percent survival of zebrafish exposed to control (E3), 0.5 mg/ml saponin, 0.7 mg/ml saponin and 1 mg/ml saponin from 6-9 dpf (*n* = 40 fish/treatment) (Log-rank Mantel-Cox Test for Chi-square, ^∗∗∗^*p* < 0.0005). **(B)** Representative pictures of the saponin-treated Tg(mpeg1:mCherry/mpx:eGFPi^114^) fish displaying green neutrophils and red macrophages. **(C)** Quantification of neutrophils and macrophages in the intestinal area (*n* = 6–11 fish/group) (one way ANOVA Kruskal-Wallis test with Dunn’s Multiple comparison Post-Test, mean ± SEM, ^∗^*p* < 0.05 ^∗∗^*p* < 0.01). Top: counted cells in intestinal area. Bottom: Corrected Total Cell Fluorescence (CTCF, measure for total fluorescent pixels in the whole fish). Two independent experiments were performed and data are combined.

Exposing zebrafish larvae to highly purified saponin (95% pure) from 6 to 9 dpf decreased the percentage of survival in a dose-dependent fashion (Figure 2A). Zebrafish mortality increased significantly at 1 mg/ml soy saponin compared to controls. Increased neutrophil recruitment to the intestinal area (region indicated in Supplementary Figure S1) increased in a dose-dependent manner at 72 but not at 24 h after exposure (Figures 2B,C). Linear regression on the intestinal neutrophil count revealed a significant dose-response to saponin immersion (Supplementary Figure S2). However, the corrected total cell fluorescence (CTCF, total corrected fluorescent signal in the green channel) in the entire fish is not increased (Figure 2C), suggesting that saponin induced intestinal-region specific effects. Interestingly, since some larvae (as the one depicted in Figure 2B) showed stronger fluorescence signal in the kidney area, corrected total cell fluorescence was also assessed in the kidney region. However, both at 24 and 72 h we did not observe a significant increase in kidney fluorescence with increasing saponin dose (data not shown). In contrast to the neutrophils, the macrophages were not affected by saponin at 0.5, 0.7 or 1.0 mg/ml doses at both 24 and 72 h (Figures 2B,C).

### Saponin Dose-Dependently Induced Pro- and Anti-inflammatory Cytokine Expression

**FIGURE 3:**
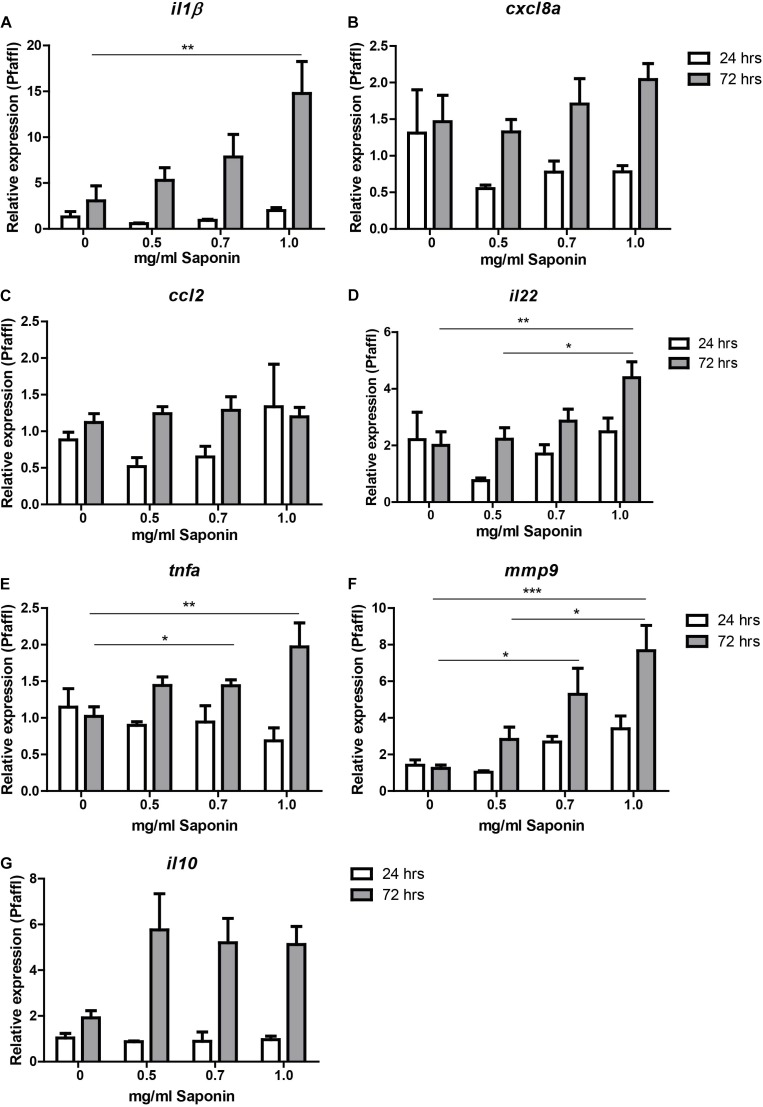
Relative gene expression of saponin-treated zebrafish. Zebrafish were immersed in different doses of saponin (0, 0.5, 0.7 and 1 mg/ml, from 6 to 9 dpf). At 24 h (7 dpf) and 72 h (9 dpf) zebrafish larvae were euthanized and 5 whole zebrafish were pooled for each sample. After RNA extraction and cDNA synthesis, qPCR was performed for the following cytokines: **(A)** *il-1*β, **(B)** *cxcl8a*, **(C)** *ccl2*, **(D)** *il22*, **(E)** *tnfa (tnfα2)*, **(F)** *mmp9*, and **(G)** *il10.* Three pools of five larvae were used per group at 24 h, and 7–9 pools of five larvae per group at 72 h. Data were tested for normality with Kolmogorov-Smirnov. Non-parametric analysis was performed by one way ANOVA Kruskal-Wallis test with Dunn’s Multiple comparison post-test for *il1b* and *mmp9.* Parametric analysis was performed by one way ANOVA with Tukey *post hoc* for *il22*, and *tnfa*. Results are displayed as mean ± SEM. Two independent experiments were performed and the data were combined.

As can be observed from Figure 3, expression of pro-inflammatory cytokine *il1b* increased significantly after immersion in 1.0 mg/ml saponin for 72 h compared to controls (Figure 3A). The expression of *tnfa* increased significantly after 72 h when larvae were exposed to a dose of 0.7 or 1.0 mg/ml of saponin (Figure 3E). Increased expression of *mmp9* (involved in breakdown of extracellular matrix, indicative of tissue damage) was seen after exposure to 0.7 mg/ml or 1.0 mg/ml saponin immersion after 72 h (Figure 3F). *Il22*, a regulatory cytokine of the il10 family, showed increased expression at 72 h after immersion in 1.0 mg/ml saponin (Figure 3D). While *il10* expression data showed a significant value for the Kruskall-Wallis test (*p* = 0.02), *post hoc* testing using Dunn’s multiple comparison did not show differences between groups (Figure 3G). The expression of both *cxcl8a* and *ccl2* did not change upon saponin exposure (Figures 3B,C).

### Zebrafish Exposed to Oxytetracycline From 4 to 6 dpf Showed Slightly Lower *il1b* Expression, However, Neutrophil Recruitment Was Not Affected

In order to address whether early exposure to antibiotics ciprofloxacin or oxytetracycline already affects zebrafish larvae at baseline, we exposed the larvae to either 5 ug/L ciprofloxacin or 5 ug/L oxytetracycline from 4 to 6 dpf. Neutrophil and macrophage recruitment to the intestinal area as measured by the number of these innate cells was not altered (Supplementary Figure S3). Furthermore, gene expression analysis revealed that oxytetracycline but not ciprofloxacin induced a small but significant decrease in *il1b* expression (Supplementary Figure S3).

### Ciprofloxacin or Oxytetracycline Did Not Reduce Saponin-Induced Neutrophil Recruitment to the Intestinal Area Upon Co-treatment

**FIGURE 4:**
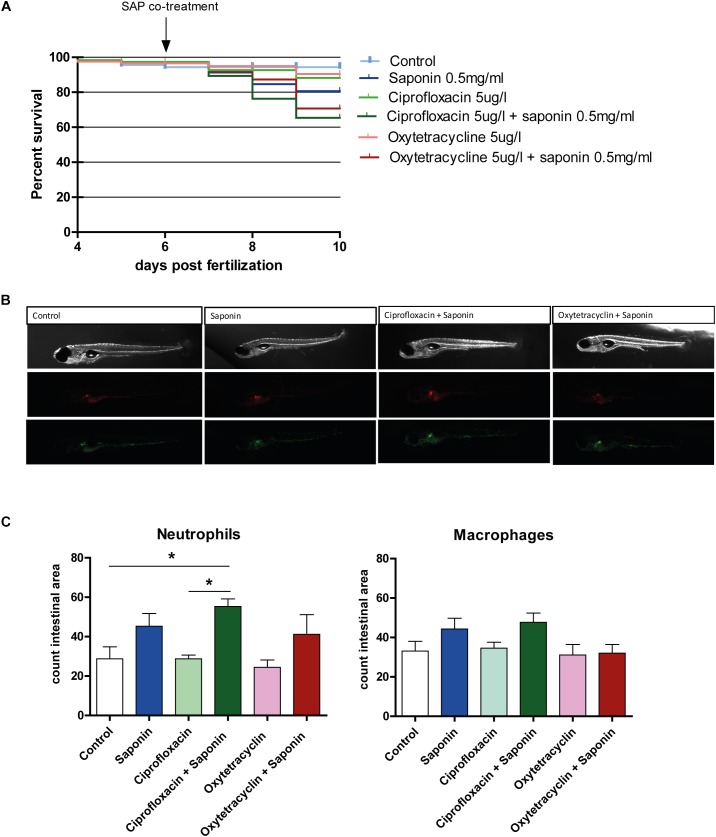
Effect of antibiotic exposure on saponin-immune-stimulation. **(A)** Percent survival of zebrafish exposed to control (E3), ciprofloxacin (4–9 dpf) (5 ug/L) or oxytetracycline (4–9 dpf) (5 ug/ml) +/- saponin (0.5 mg/ml) from 6 – 9 dpf (*n* = 100 fish/treatment) (Log-rank Mantel-Cox Test for Chi-square). **(B)** Representative pictures of the antibiotic/saponin-treated Tg(mpeg1:mCherry/mpx:eGFPi^114^) fish displaying green neutrophils and red macrophages. **(C)** Quantification of neutrophils and macrophages in the intestinal area (*n* = 10 fish/group) (one way ANOVA Kruskal-Wallis test with Dunn’s Multiple comparison Post-Test, mean ± SEM, ^∗^*p* < 0.05). Two independent experiments were performed and one representative experiment is shown.

To understand whether antibiotics protected from or enhanced the saponin-induced immune stimulation, we exposed the fish to either oxytetracycline or ciprofloxacin (4–9 dpf) in the presence or absence of a low dose (0.5 mg/ml) of saponin immersion (6–9 dpf). We specifically chose this dose of 0.5 mg/ml to induce mild (sub-phenotypical) immune activation, so to mimic low amounts of anti-nutritional factors. We recorded mortality to assess cytotoxicity derived from saponin and ciprofloxacin/oxytetracycline exposure (Figure 4A). All saponin treated groups showed a lower, but not significant, survival compared to controls or antibiotics alone.

**FIGURE 5:**
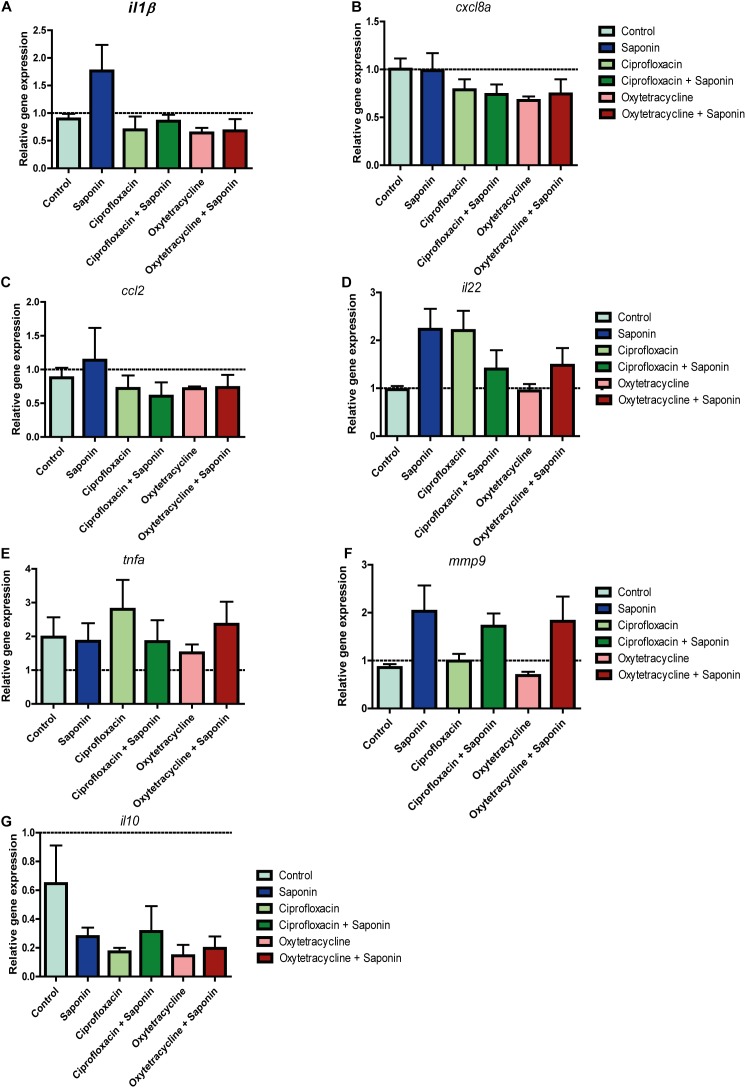
Relative gene expression of antibiotic/saponin-treated zebrafish. At 72 h after exposure (9 dpf) zebrafish larvae were euthanized and 5 whole zebrafish were pooled for each sample. After RNA extraction and cDNA synthesis, qPCR was performed for the following cytokines: **(A)** *il-1*β, **(B)** *cxcl8a*, **(C)** *ccl2*, **(D)** *il22*, **(E)** *tnfa (tnfα2)*, **(F)** *mmp9*, and **(G)** *il10.* (*n* = 4 pools of 5 larvae/treatment, one way ANOVA Kruskal-Wallis test with Dunn’s Multiple comparison Post-Test, mean ± SEM).

As can be observed from Figures 4B,C, the combination of ciprofloxacin and low-dose saponin significantly increased neutrophil recruitment to the intestinal area. Exposure to only saponin or the combination of oxytetracyclin and saponin only showed a trend toward increased neutrophil presence in the intestinal area (*p* < 0.10). Antibiotic treatment alone or in combination with saponin did not show significant changes in gene expression. However, *il1b*, *cxcl8*, and *il22* genes expression all tended to be lower in antibiotic co-treatment, with the exception of *mmp9* that tended to be higher in groups receiving saponin (Figures 5A,B,D,F). The expression of *ccl2*, *tnfa*, and *il10* was not different between treatment groups (Figures 5C,E,G).

### Combination of Oxytetratcycline and Saponin Significantly Increased Microbiota Diversity

**FIGURE 6:**
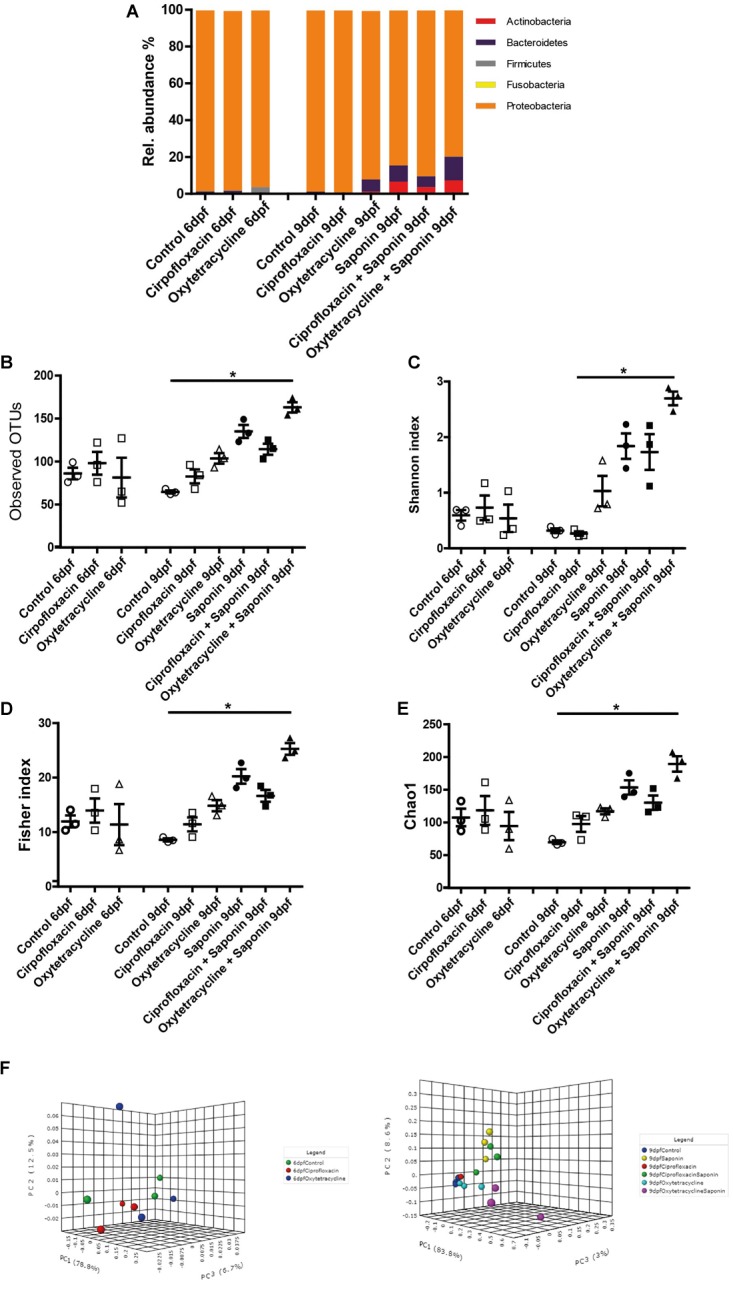
Saponin altered the microbiome and co-exposure with oxytetracycline increased microbiota diversity. **(A)** Relative abundance in microbiota at phylum level for the treatment groups. Several richness and α-diversity indexes were analyzed: **(B)** Observed OTUs (richness), **(C)** Shannon’s diversity index, **(D)** Fisher index, **(E)** Chao1 index (*n* = 3 pools of 5 larvae/treatment) (one way ANOVA Kruskal-Wallis test with Dunn’s Multiple comparison Post-Test, mean ± SEM, ^∗^*p* < 0.05,). β-diversity PCoA plots are displayed for 6dpf (baseline) **(F)** and 9 dpf (end of the treatments exposure) **(G)** (Statistics: PERMANOVA).

Assessment of the microbiota composition at phylum level showed that Proteobacteria was the most abundant phylum observed (Figure 6A). In all groups receiving saponin, the relative abundance of Bacteroidetes and Actinobacteria seemed increased, however, these changes were not significantly different from the 9 dpf control. Interestingly, this trend in Bacteroidetes and Actinobacteria was also observed in the fish that received oxytetracycline only. The increase in diversity richness was further confirmed by the observed OTUs (Figure 6B) and the α-diversity indexes (Figures 6C–E). The combination of saponin and oxytetracycline displayed a significant increase in α-diversity (Shannon, Chao and Fisher) compared to control at 9 dpf (Figures 6C–E). In order to assess β-diversity we performed Principal Coordinate Analysis (PCoA) (Figures 6F,G). At 6 dpf, clustering of each treatment (R2: 0.23, *p*-value < 0.53) did not reveal a significant relationship whereas at 9 dpf (R2: 0.86, *p*-value < 0.001) both saponin and antibiotic treatment were the major determinant for the variation between the microbial communities.

### Redundancy Analysis Revealed That Saponin Promoted a Microbial Shift Which Was Further Enhanced by Oxytetracycline

**FIGURE 7:**
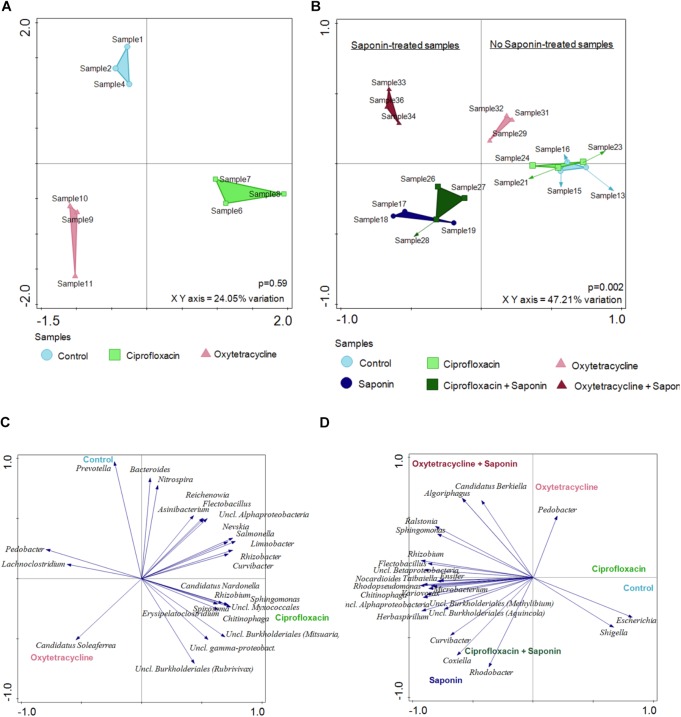
Saponin promoted a microbiome shift which was further enhanced by oxytetracycline in zebrafish larvae. Redundancy Analysis (RDA) was performed at 6dpf **(A)** and at 9 dpf **(B)** (*n* = 3 pools of 5 larvae/treatment). The distances among the samples approximates the average dissimilarity of the genera composition between the two samples being compared as measured by Euclidean distances. The top 25 most discriminating (not *per se* most abundant) OTUs are displayed at **(C)** 6 dpf and **(D)** 9 dpf for all the treatments.

To get more insight into the microbiome shift upon saponin addition a Redundancy Analysis (RDA) was performed at 6 dpf (basal level, Figure 7A) and at 9 dpf (end of the treatment, Figure 7B). At basal level (6 dpf) the samples clustered by treatment depending on the top 25 most discriminating OTUs. However, those differences were not significant and the X and Y axis can just explain 24.05% of the variation observed. On the other hand, at 9 dpf, after saponin addition, the shift was substantial. The treatments clustered separately among saponin-treated groups and non-saponin-treated groups. Differences were significant (*p* = 0.002) and the X and Y axis accounted for 47.21% of the variation observed. The top 25 most discriminating (not *per se* most abundant) OTUs are displayed in Figure 7C (6 dpf) and Figure 7D (9 dpf) and correlated with the treatment groups. Importantly, the angle between the genus and the imaginary line from the treatment to the (x = 0, y = 0) coordinate displays the correlation among genus and treatment: i.e., 5° are strongly correlated, 90° are not correlated, 180°are inversely correlated. Therefore, the genus *Escherichia* and *Shigella* were correlated with control group while *Curvibacter*, *Coxiella* and *Rhodobacter* were associated with a saponin and saponin + ciprofloxacin-treated group. On the other hand, *Pedobacter* was correlated with oxytetracycline-treated fish while *Candidatus berkiella* and *Algoriphagus* were associated with oxytetracycline + saponin-treated fish. Interestingly, the oxytetracycline-treated groups differed from the ciprofloxacin and the control groups (with and without saponin), indicating that the microbiome shift was saponin but also antibiotic treatment dependent.

### The Diversity of Several Genera of Bacteria Increased at the Expense of Escherichia After Saponin and Oxytetracycline Exposure

**FIGURE 8:**
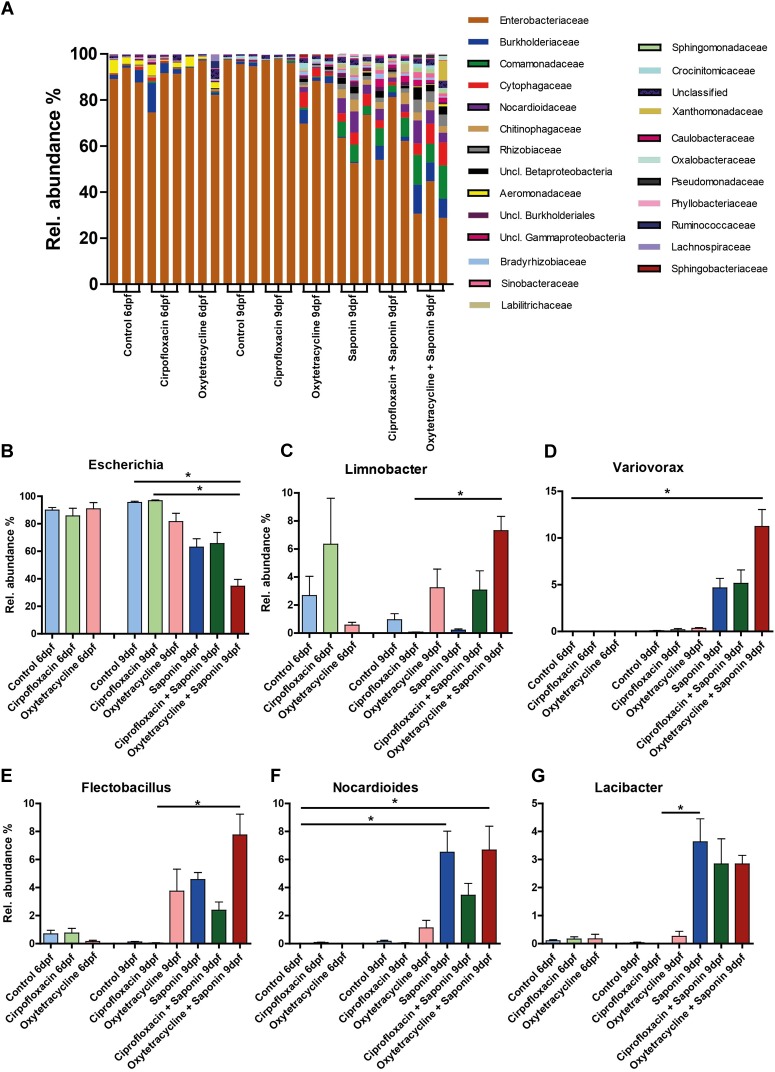
Diversity of several bacterial genera increased at the expense of *Escherichia* after saponin and oxytetracycline exposure. **(A)** The overall average ≥ 0.05% abundant genera are displayed (3 pools of 5 larvae/treatment). The top 6 most abundant genera are displayed: **(B)** *Escherichia*, **(C)** *Limnobacter* **(D)** *Variovorax* **(E)** *Flectobacillus* **(F)** *Nocardioides*, **(G)** *Lacibacter* (mean ± SEM, ^∗^*p* < 0.05).

To assess the weight of individual genera the overall average ≥ 0.05% abundant genus was assessed and the top 6 abundance are depicted in Figure 8 (top 7 – 10 can be found in Supplementary Figure S4). Strikingly, *Escherichia* was the most abundant bacterium in every sample. However, a significant reduction of *Escherichia* was observed in the oxytetracycline + saponin-treated fish compared to the controls (Figure 8B). Changes were observed in *Limnobacter* (Figure 8C), *Variovorax* (Figure 8D), *Flectobacillus* (Figure 8E), *Nocardioles* (Figure 8F) and *Lacibacter* (Figure 8G). All tended to be more abundant in the saponin treated groups, while *Limnobacter* and *Flectobacillus* also tended to be higher in the oxytetracyclin alone group. *Aeromonas* (Supplementary Figure S4) was found at 6 dpf but not at 9 dpf suggesting that this genus is related to earlier life stages in our zebrafish larvae.

## Discussion

In this study we investigated whether environmental levels of antibiotics in the water could influence zebrafish larval immune responses toward sub-phenotypical levels of anti-nutritional factors. We found that immersion in saponin induced immune stimulation in a dose dependent manner (as measured by neutrophil influx to the intestinal region and pro-inflammatory cytokine responses in the whole animal). Combined exposure to ciprofloxacin and saponin increased neutrophil influx to the gut area significantly compared to controls and exposure to either saponin or ciprofloxacin alone. Moreover, immersion in saponin combined with oxytetracycline significantly increased the diversity of the microbiota: the abundance of Bacteroidetes and Actinobacteria increased at the expense of Proteobacteria. Beta-diversity analysis revealed that the treatments (antibiotics and saponin) were microbial shift determining factors.

Saponins contain one carbohydrate chain linked to a fat soluble region and they are able to disrupt biological membranes due to their amphipathic nature. Several studies show that saponins are the main anti-nutritional factor in soybean meal causing intestinal inflammation in fish species (Chikwati et al., 2012; Costas et al., 2014; Krogdahl et al., 2015). As already shown by Hedrera et al. (2013) saponin is able to induce an immune response in zebrafish at 3.3 g/kg inclusion level in feed. Here, it is important to note that levels of saponins within soybean meal can differ greatly. In this study, as in the study by Hedrera highly pure sources of saponin (95 and 90%, respectively) have been used which ameliorate the reproducibility of the results. An important difference between our study and the study by Hedrera is that in this study the saponin was supplied to the water and not incorporated into the feed. Therefore, our data yield information on the response of the whole fish, and not necessarily on intestinal specific effects. We did observe that the number of neutrophils present in the intestinal area does increase dose-dependently upon saponin immersion. A dose-dependent increase in whole fish neutrophil fluorescence (as measured by corrected total cell fluorescence, CTCF) was not observed. Likewise, the CTCF originating from neutrophils in the (head)kidney area in larvae treated with saponin was also not increased upon higher saponin dose. The fact that increased neutrophil presence is observed in our study in the gut area might be partly explained by the fact that the larvae are fed Tetrahymena throughout the study from 5 dpf. Therefore, the surrounding water containing saponin is ingested together with the feed, and saponin will reach the larval intestines. Although, the aquaculture fish industry has almost fully replaced soybean meal and saponin in the feed, this saponin-induced immune stimulation model can be used as a screening method to assess other compounds for their ability to cause comparable immune stimulation, or their ability to protect from inflammation.

In the present study, a dominance of Proteobacteria in our control larvae was observed, which is in line with much of the literature (Roeselers et al., 2011; Sullam et al., 2012; Stephens et al., 2016). However, the dominant Proteobacteria observed in our larvae was *E. coli*, which is not often found in other studies. Exposure to saponin in the rearing water, shifted the entire microbiome and tended to increase its diversity (as indicated by the observed OTUs and α-diversity). We hypothesize that supplying an additional substrate (saponin) in a very short period, from 6–9 dpf, next to the live feed (tetrahymena) might have just favored other less abundant species from the Bacteroidetes and Actinobacteria phyla to grow out at the expense of the dominant Proteobacteria. This might be different when older zebrafish with a more diversified and stable microbiota would have been used. An increase of Bacteroidetes was also reported in 30 dpf zebrafish fed a high fat diet, resulting in an altered microbiome compared to controls (Arias-Jayo et al., 2018). However, in the study of Arias-Jayo and colleagues fish were fed with a commercially available pellet feed during 25 days 3 times per day and therefore we cannot compare it with the settings of our study.

Interestingly, fish that were only exposed to oxytetracycline tended to show increased microbial diversity. This is not in line with current literature, in which most studies investigating antibiotics observe reduced diversity (Navarrete et al., 2008; Ding and He, 2010; Pindling et al., 2018; Zhou et al., 2018a,b). The fact that saponin (and soybean meal) increases bacterial diversity in fish is observed by others (Bakke-McKellep et al., 2007; Merrifield et al., 2009). Furthermore, in our study we have used very young larvae (4–9 dpf) in which the microbial community is still developing and that at this stage the microbiota of zebrafish larvae greatly resembles the environmental microbiota (Stephens et al., 2016). Investigating effects of antibiotics and saponin exposure to older fish, with a more diversified and stable microbiota might give different results.

Oxytetracycline by itself also tended to increase diversity and had some effects on baseline gene expression; significantly reducing *il1b* expression (Supplementary Figure S3). Furthermore, ciprofloxacin and oxytetracycline affect microbes differently; ciprofloxacin mainly inhibits DNA synthesis and replication of aerobic Gram-negative bacteria, while oxytetracycline inhibits protein synthesis of both anaerobic Gram-positive as well as Gram-negative bacteria (Perrin-Guyomard et al., 2005; Ding and He, 2010). Understanding the effect of these antibiotics not only on the presence but also transcriptional activity of microbes associated to a fish host might be very interesting to identify why oxytetracycline has other effects on the host compared to ciprofloxacin.

Our study is in line with a previous study performed in Atlantic Salmon where oxytetracycline was added at 3 g/kg to soybean meal diet. The oxytetracycline did not affect disease severity, however it did influence the microbial community (Bakke-McKellep et al., 2007). Considering the fact that we used low levels of oxytetracycline and ciprofloxacin, far lower than the levels that can be found at aquaculture sites during treatment (Ding and He, 2010; Plhalova et al., 2014; Carvalho and Santos, 2016), it would be very interesting to follow especially the oxytetracycline-exposed fish to older age and assess their disease susceptibility. Zhou et al. (2018a) fed adult zebrafish oxytetracycline-containing feed for 6 weeks and observed that these fish displayed higher mortality upon *Aeromonas* challenge as well as displayed lower activity of alkaline phosphatase and acid phosphatase needed for intestinal homeostasis (Bates et al., 2007).

## Conclusion

In conclusion, in this study we have shown that saponin immersion dose-dependently induces immune stimulation, as evidenced by increased pro-inflammatory cytokine expression and neutrophil recruitment to the intestinal area. Low levels of antibiotics present in surface water can influence saponin-induced changes in the microbiome (increased α-diversity in oxytetracycline + saponin) and increased neutrophil recruitment (ciprofloxacin + saponin). Therefore, this study highlights the importance of background levels of environmental pollutants such as antibiotics in the assessment of feed effects on fish health, which may be missed in controlled laboratory settings.

## Author Contributions

AL performed the experiments and drafted the manuscript. DP advised on the experiments and wrote the manuscript. GW wrote the manuscript and provided the funding. SB performed the experiments, wrote the manuscript and provided the funding.

## Conflict of Interest Statement

The authors declare that the research was conducted in the absence of any commercial or financial relationships that could be construed as a potential conflict of interest.

## Supplementary Material

FIGURE S1 Representative picture of the intestine region selected in the bright field and copied to the green and red channel pictures for neutrophil and macrophage count.

FIGURE S2 Linear regression analysis of the neutrophil count in the intestinal area of the saponin dose-response experiment.

FIGURE S3 Representative pictures of neutrophil and macrophage imaging at baseline (6 dpf) for control (E3), Ciprofloxacin and Oxytetracycline treated larvae (exposure from 4–6 dpf), **(B)** Quantification of neutrophil and macrophage number in the intestinal area (*n* = 10 larvae/treatment). **(C)** Relative gene expression on pools of whole zebrafish larvae (*n* = 4 pools of 5 larvae/treatment) for the following cytokines: *il-1*β, *ccl2, cxcl8*, (one way ANOVA Kruskal-Wallis test with Dunn’s Multiple comparison Post-Test, mean ± SEM.

FIGURE S4 The top 7–10 most abundant genera (mean ± SEM, ^∗^*p* < 0.05) are displayed: **(A)** Unclassified *Betaproteobacteria* **(B)** *Aeromonas* **(C)** *Rhizobium* **(D)** Unclassified *Gammaproteobacteria.*
