# Corrigendum: Exposure to Antibiotics Affects Saponin Immersion-Induced Immune Stimulation and Shift in Microbial Composition in Zebrafish Larvae

**DOI:** 10.3389/fmicb.2019.01242

**Published:** 2019-06-03

**Authors:** Adrià López Nadal, David Peggs, Geert F. Wiegertjes, Sylvia Brugman

**Affiliations:** ^1^Cell Biology and Immunology, Animal Sciences Group, Wageningen University and Research, Wageningen, Netherlands; ^2^Skretting Animal Research Centre, Stavanger, Norway; ^3^Aquaculture and Fisheries, Animal Sciences Group, Wageningen University and Research, Wageningen, Netherlands

**Keywords:** saponin, zebrafish, microbiota, oxytetracycline, ciprofloxacin, neutrophils, macrophages

In the original article, there was an error. The name of the transgenic line used was incorrect. The correct name of the line is “mpeg1:mCherry/mpx:eGFPi^114^”

Corrections have been made to the **Materials and Methods** subsection **Animals**:

“Adult Tg(mpeg1:mCherry/mpx:eGFPi^114^) (Renshaw et al., 2006; Bernut et al., 2014) zebrafish (kindly provided by Prof. Meijer, Leiden University), expressing mCherry under the macrophage-specific mpeg1 promotor and GFP under the neutrophil-specific mpx promotor were housed in Zebtec family tanks (Tecniplast, Buguggiate, Italy) under continuous flow-through at 28°C (14/10-hour light/dark cycle) at Carus facilities (WUR, Wageningen, Netherlands). Zebrafish were fed with a mixture of Artemia 230.000 npg (Ocean Nutrition Europe, Essen, Belgium) and Tetramin Flakes (Tetra, Melle, Germany) twice per day. Embryos were obtained by natural spawning and raised with E3 water (0.10 mM NaCl in demineralized water, pH 7.6) in petri dishes at 28°C (12/12-hour light/dark cycle) (Westerfield, 2007). Dead or fungus-infected embryos were identified by microscopy and discarded in tricaine/E3 solution [8.4% (v/v) 24 mM Tricaine (Sigma-Aldrich, DL, United States) stock solution in E3]. Larval ages are expressed in days post-fertilization (dpf). From 5 dpf onward larvae were fed with live daily cultured *Tetrahymena pyriformis*.”

**Materials and Methods**, subsection **Dose-Response Experiment Saponin Exposure**:

“Double Tg(mpeg1:mCherry /mpx:eGFPi^114^) zebrafish larvae were randomly distributed in 6 well plates (*n* = 20 fish/well) and exposed to different concentrations [0, 0.5, 0.7 and 1.0 mg/ml] of saponin [ultrapure Soy Saponin 95%, kindly provided by Trond Kortner NMBU Oslo Norway, origin: Organic Technologies, Coshocton, OH (Krogdahl et al., 2015)] dissolved in the E3 (10 ml solution/well) from 6–9 dpf. Mortality was registered and all media were refreshed daily. At 24 h (7 dpf) and 72 h (9 dpf) after the start of the immersion, zebrafish (*n* = 6–11/group) were anaesthetized embedded and imaged using fluorescent microscopy (as described below). Per time point several larvae were euthanized for further analysis with an overdose MS-222 (8.4 ml of 24 mM Tricaine (Sigma-Aldrich, DL, United States) in 100 ml E3). Pools of 5 larvae were used for RNA extraction (3 pools per group at 24 h, 7–9 pools per group at 72 h) and gene expression was measured on cDNA by Real Time PCR (as described below). Two independent experiments were performed and data were combined.”

**Materials and Methods**, subsection **Fluorescent** ***in vivo* imaging**:

“Tg(mpeg1:mCherry/mpx:eGFPi^114^) zebrafish larvae were anaesthetized with tricaine/E3 solution (4.2 ml of 24 mM Tricaine (Sigma-Aldrich, DL, United States) in 100 ml E3) and embedded in 1% low melting point agarose (Thermo Fisher Scientific, MA, United States). Larvae were imaged as whole mounts with a Leica M205 FA Fluorescence Stereo Microscope. After image acquisition, pictures were analyzed with ImageJ® software (United States National Institutes of Health, Bethesda, United States). The intestinal regions were manually selected per fish on the basis of the bright light picture and subsequently copied to the green and red channel pictures (Supplementary Figure S1). Within this intestinal region individual cells were counted for each fish. Furthermore, corrected total cell fluorescence (CTCF) was measured in ImageJ® on total fish larvae by using the following formula: Integrated density—(area of total fish x mean fluorescence of the background reading).”

**Material and Methods**, subsection **Experimental Design and Sampling Strategy Antibiotics and Saponin Exposure**:

A graphical representation of the experimental design and analysis performed per time-point is displayed in Figure 1. To assess the effect of antibiotics, 4 dpf Tg(mpeg1:mCherry/mpx:eGFPi^114^) fish were randomly distributed in five 6 well-plates (*n* = 20 fish/well) and 3 treatment conditions were established: (1) control (E3), (2) ciprofloxacin 5 μg/L (Sigma-Aldrich, DL, United States) or (3) oxytetracycline hydrochloride 5 μg/L (Sigma-Aldrich, DL, United States) (10 ml solution/well). The dose of antibiotics was based on several reviews and experimental papers summarizing environmental concentrations of antibiotics in water environments (Ding and He, 2010; Carvalho and Santos, 2016; Watts et al., 2017; Patrolecco et al., 2018; Zhou et al., 2018b) to be at a low dose (ng-μg/L range) and not acute dose (mg/L range). At 6 dpf, 4 pools of 5 larvae were sampled to assess changes in gene expression at baseline. Moreover, at 6 dpf DNA was isolated from 3 pools of 5 larvae to investigate microbiome composition at baseline. *In vivo* imaging was performed on *n* = 10 larvae/group to visualize innate immune cells. Subsequently, after sampling, at 6 dpf ultrapure soy saponin was applied to half of the remaining larvae at a concentration 0.5 mg/ml (to induce mild immune stimulation) so each treatment group was split into two, resulting in 6 treatment groups: (1) control, (2) ciprofloxacin (5 μg/L), (3) oxytetracycline hydrochloride (5 μg/L), (4) saponin (0.5 mg/ml), (5) ciprofloxacin + saponin (5 μg/L + 0.5 mg/ml), and (6) oxytetracycline hydrochloride + saponin (5 μg/L + 0.5 mg/ml). All treatment media were refreshed daily. At 9 dpf *in vivo* imaging was performed on *n* = 10 larvae/group to visualize innate immune cells. Gene expression was performed on 4 pools of 5 larvae to investigate immune gene expression and from 3 pools of 5 larvae DNA was isolated for microbiological analysis.

Because of the error reported above, corrections have also been made to the Figure legends of Figure 2 and Figure 4. The correct legends appear below.

**Figure 2**: Effect of saponin immersion on zebrafish larvae. **(A)** Percent survival of zebrafish exposed to control (E3), 0.5 mg/ml saponin, 0.7 mg/ml saponin and 1 mg/ml saponin from 6–9 dpf (*n* = 40 fish/treatment) (Log-rank Mantel-Cox Test for Chi-square, ***p < 0.0005). **(B)** Representative pictures of the saponin-treated Tg(mpeg1:mCherry/mpx:eGFPi^114^) fish displaying green neutrophils and red macrophages. **(C)** Quantification of neutrophils and macrophages in the intestinal area (*n* = 6–11 fish/group) (one way ANOVA Kruskal-Wallis test with Dunn's Multiple comparison Post-Test, mean ± SEM, *p < 0.05 **p < 0.01). Top: counted cells in intestinal area. Bottom: Corrected Total Cell Fluorescence (CTCF, measure for total fluorescent pixels in the whole fish). Two independent experiments were performed and data are combined.

**Figure 4**: Effect of antibiotic exposure on saponin-immune-stimulation. **(A)** Percent survival of zebrafish exposed to control (E3), ciprofloxacin (4–9 dpf) (5 ug/L) or oxytetracycline (4–9 dpf) (5 ug/ml) + /– saponin (0.5 mg/ml) from 6–9 dpf (n = 100 fish / treatment) (Log-rank Mantel-Cox Test for Chi-square). **(B)** Representative pictures of the antibiotic/saponin-treated Tg(mpeg1:mCherry/mpx:eGFPi^114^) fish displaying green neutrophils and red macrophages. **(C)** Quantification of neutrophils and macrophages in the intestinal area (*n* = 10 fish/ group) (one way ANOVA Kruskal-Wallis test with Dunn's Multiple comparison Post-Test, mean ± SEM, *p < 0.05). Two independent experiments were performed and one representative experiment is shown.

The authors apologize for this error and state that this does not change the scientific conclusions of the article in any way. The original article has been updated.
