# Supplementary material for: Exposure to Antibiotics Affects Saponin Immersion-Induced Immune Stimulation and Shift in Microbial Composition in Zebrafish Larvae
Source: Front Microbiol. 2018 Oct 29;9:2588. doi: 10.3389/fmicb.2018.02588 (PMC6215861; doi:10.3389/fmicb.2018.02588)

Supplementary figure 1, Lopez Nadal *et al.*

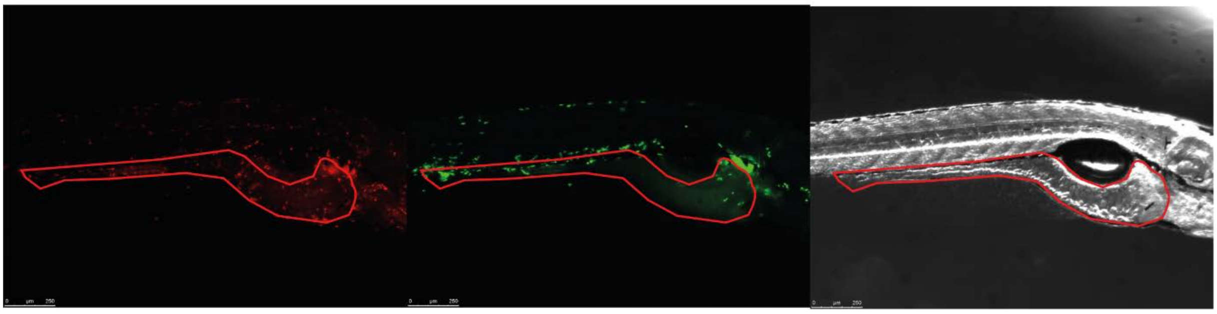

Supplementary figure 2, Lopez Nadal *et al.*

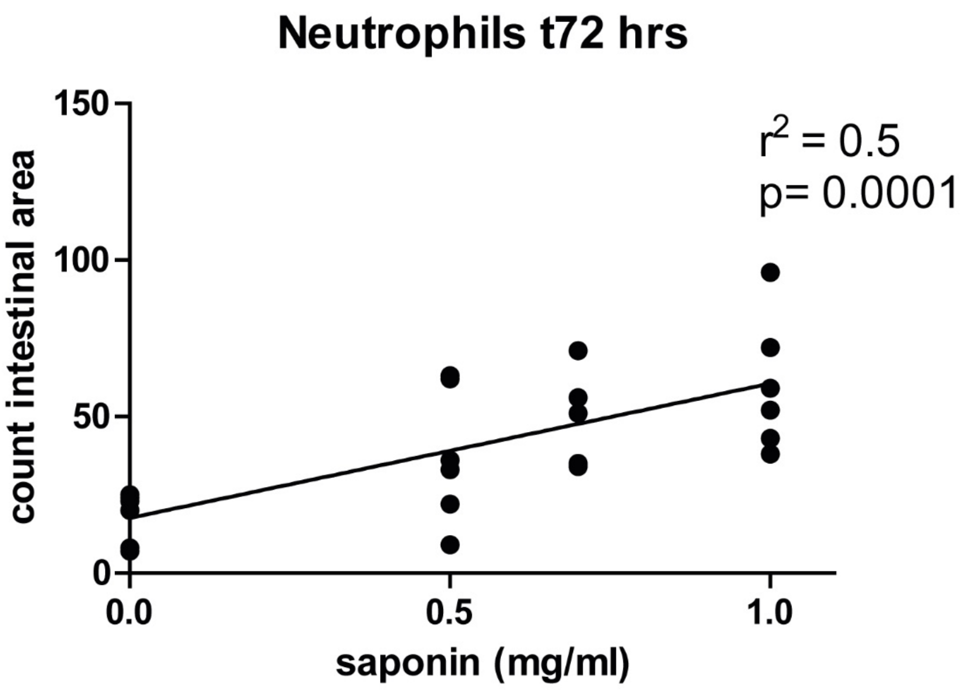

Supplementary figure 3, Lopez Nadal *et al*.

A

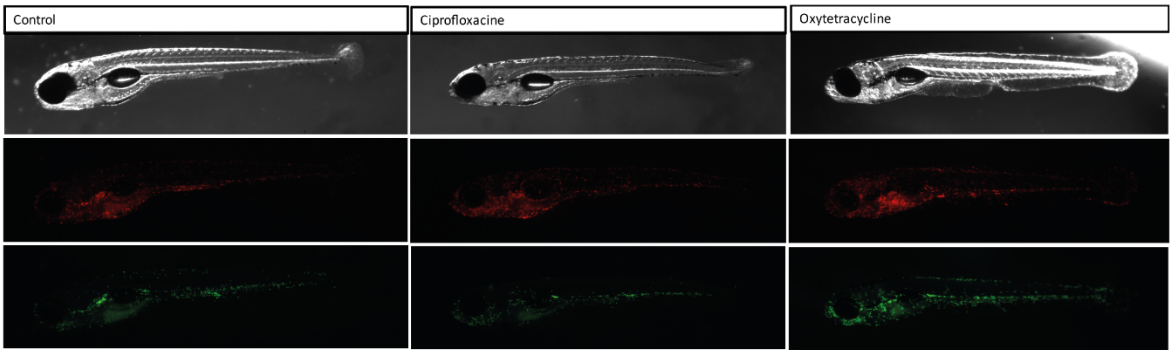

B

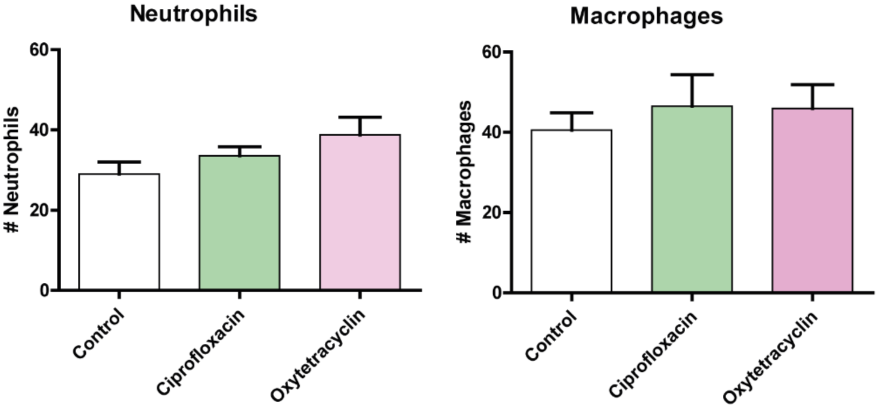

C

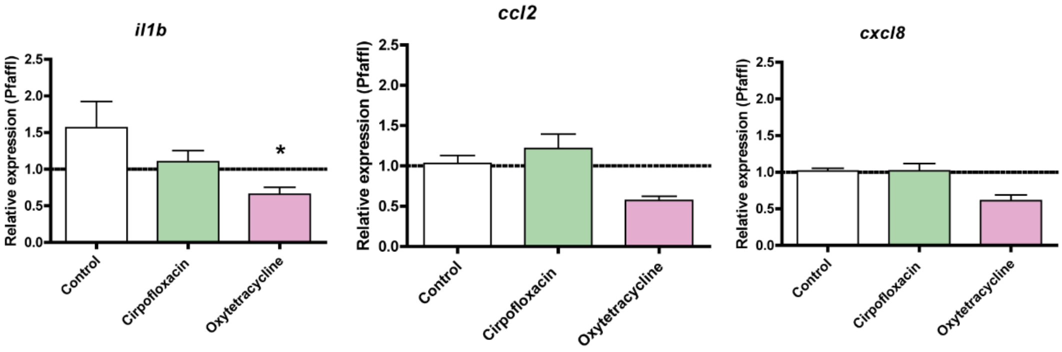

Supplementary figure 4, Lopez Nadal *et al.*

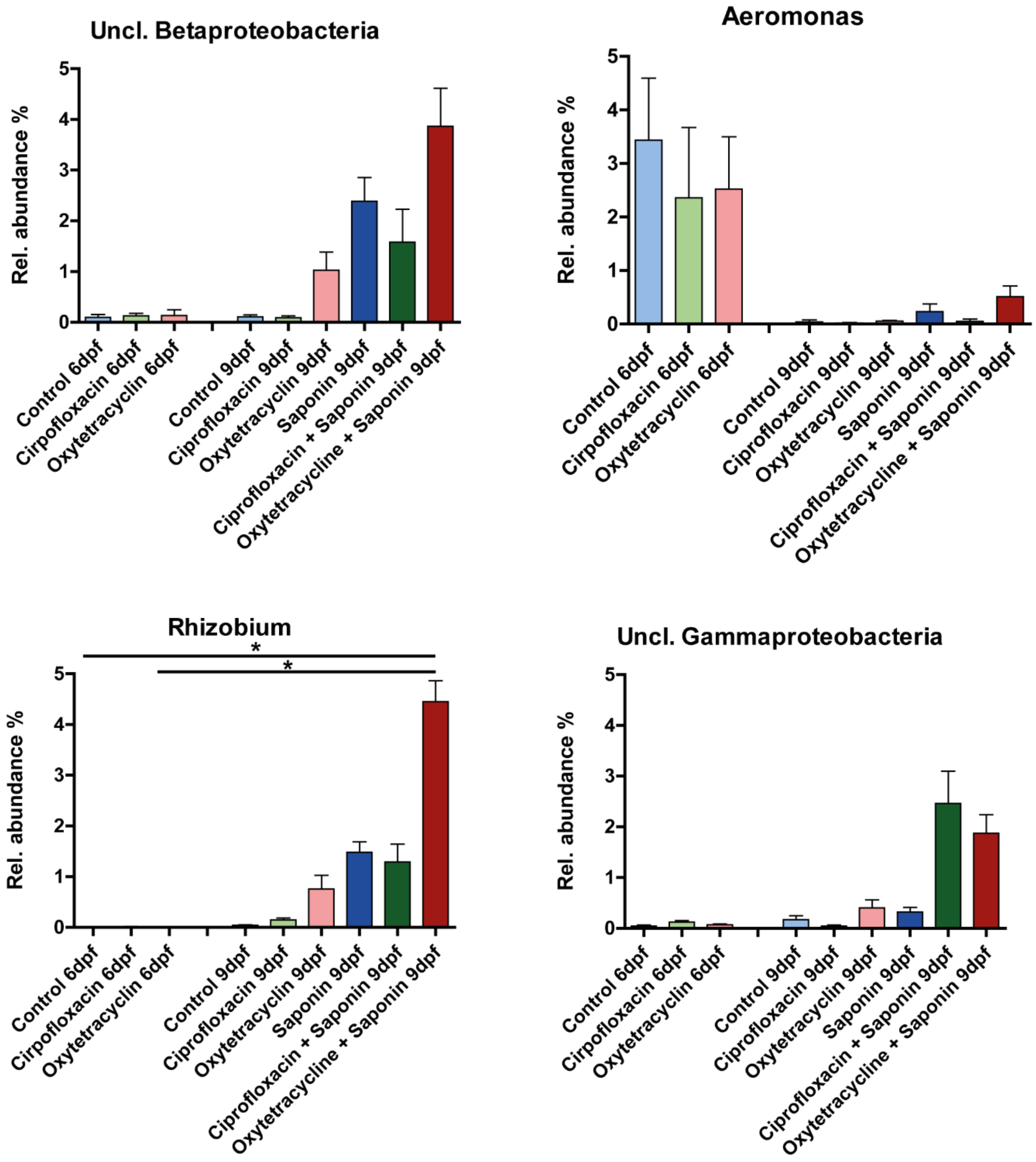

Supplement: FIGURE S1 — Representative picture of the intestine region selected in the bright field and copied to the green and red channel pictures for neutrophil and macrophage count. [file Data_Sheet_1.PDF]
